# Survival of Coliform Bacteria in Virgin Olive Oil

**DOI:** 10.1155/2018/8490614

**Published:** 2018-11-27

**Authors:** Biagi Angelo Zullo, Lucia Maiuro, Gino Ciafardini

**Affiliations:** Department of Agricultural, Environmental and Food Sciences, University of Molise, Via De Sanctis, 86100 Campobasso, Italy

## Abstract

Coliform bacteria consist of both nonpathogen commensal and human opportunistic pathogen species isolated from different habitats like animals, man, vegetables, and water. Olives normally carry natural nonpathogenic epiphytic bacteria, but during growth, harvest, and processing, one of the final products, represented by virgin olive oil, can be contaminated with coliform. Present study showed that coliform bacteria can survive and reproduce in virgin olive oil containing low level of phenolic compounds. The laboratory inoculation trials demonstrated that when the bacterium* Escherichia coli,* isolated from the olives carposphere, was transferred in olive oil containing high polar phenols content, equal to 372 mg caffeic acid equivalent per kg, the survival was completely inhibited after 15 days of storage. On the contrary, the bacterium reproduced quickly when it was inoculated in virgin olive oil samples containing lower concentration of polar phenols. The SDS-PAGE analysis of the* E. coli* proteins showed different electrophoretic patterns when the bacterium was inoculated in the virgin olive oil with high phenolic compounds content, confirming the strong interaction between the olive oil phenols content and the bacterial wall proteins. The SEM ultrastructural observations confirmed the presence of a more higher number of damaged microbial cells in virgin olive oil rich of polar phenols. This finding needs further studies since, in an era of antibiotic resistance, the development of new strategies to fight unwanted food bacteria is promising way for the future.

## 1. Introduction

Olive oil is one of the basic components of the Mediterranean diet which can also be found in other geographical areas where it is known for its high dietetic and nutritional value and its varied sensory characteristics. The presence of microorganisms in olive oil has been demonstrated in recent research carried out mainly on the survival of some species of yeast [1]. However, our current knowledge of the process that regulates the settlement of microorganisms in extra virgin olive oil is rather limited. In fact, it is well known that microorganisms persist for long periods in the olives' carposphere, both during the phase of development and during the ripening of the fruit attached to the plant. This occurs both in the later stages, when the fruits are processed as table olives or when they are ready for oil extraction in the mills. Currently, we do not know if the bacterial fraction of the microbiota of the olives, represented by the Enterobacteriaceae family, like the coliform bacteria, is destroyed during the extraction process in the oil mill or in the newly produced olive oil during its storage. Olives normally carry natural nonpathogenic epiphytic bacteria, but, during growth, harvest, transportation, and further processing in the mills, they can be contaminated with pathogens from animal and human sources. Contamination can arise through treating soil with organic fertilizers, such as sewage sludge, manure, and from irrigation water, as well as from pathogens which are able to persist and proliferate in vegetables [2, 3]. The coliform bacteria may represent a risk factor for consumer health and therefore a food safety problem, since some species are considered human pathogenic opportunists [4]. Coliform bacteria consist of both nonpathogen commensal and pathogen species. Many studies demonstrate that phenolic compounds, such as those widely present in virgin olive oil, have a marked biocide effect on the human opportunistic pathogen yeast species as well as on bacteria [5, 6]. Medina et al. showed that, contrary to virgin olive oil, sunflower oil and corn oil where the polyphenols are absent did not show any antimicrobial activity [7]. In a similar study, extra virgin olive oil from several Turkish regions, characterized by a total polyphenol content of between 159.99 and 189.64 mg per kg, showed antibacterial activity against* Escherichia coli,* whereas refined olive oil with a low phenolic content did not cause any significant effect [8]. These findings demonstrated that the antibacterial effect of phenolic compounds is highly correlated with the content of total polyphenols found in each type of olive oil. However, the limit of the concentration below which the antibacterial effect of the olive oil polyphenols disappears is unknown. In general, the level of the total polyphenols in virgin or refined olive oil can vary from 0 to 800 mg per kg or more; usually in virgin olive oil the range is between 100 and 400 mg of caffeic acid equivalent per kg. Olive oil is categorized as being low, medium, and high in polyphenols when their level is less than 100, between 100 and 300 and more than 300 mg of caffeic acid equivalent per kg, respectively. Considering that the current knowledge on the survival of the coliform bacteria in the oil mills during the extraction process as well as in the olive oil characterized by a low or medium level of polyphenols content is very scarce, the phenols-poorly olive oils of low quality are deemed safe for human consumption only by analogy with those of good quality [9]. On the basis of the above considerations, we studied the presence of coliform bacteria in the oil mills during the extraction process as well as the survival of* E. coli* artificially inoculated in to virgin olive oil with different total polar phenols content.

## 2. Materials and Methods

### 2.1. Sampling through the Olive Oil Extraction Process

The trials were carried out using olives, paste, and olive oil, produced in three oil mills located, respectively, in three different areas of Northern Italy (Liguria region) during the 2015 olive oil yield production. The olives of the Lavagnina, Leccino, and Taggiasca variety, produced and processed, respectively in each areas of the Liguria region, were collected at the beginning of the ripening period as aforementioned by Ciafardini and Zullo [10, 11]. The fruits were processed under typical conditions following the methods of Ciafardini et al. [12]. In detail, during the extraction process, two samples of wash water, kneaded paste and olive oil, were taken, respectively, from each mill and olive variety, using 1 L sterile-plastic containers. Half of the samples were immediately subjected to a microbiological analysis, while the remaining samples were stored at -20°C and subsequently used for chemical analysis. The olive oil routine chemical analyses were assessed according to the European Commission Regulation 640/2008 of the European Community [13]. The total polar phenols were extracted three times from kneaded paste or olive oil with a methanol:water (60:40, v/v) mixture. The Folin-Ciocalteu's phenol reagent (Merck) was added to a suitable aliquot of the combined extracts and the absorbance of the solution at 765 nm was evaluated after 1 h of incubation using a Jenway 6300 spectrophotometer (UK). Values are given as mg of caffeic acid per kg of oil.

### 2.2. The Microbiological Analysis

The microbiological analysis was carried out on the products obtained in the oil mills during the extraction process of the Lavagnina, Leccino, and Taggiasca olive varieties. Samples of olive washing water, kneaded paste, and the extracted olive oil were analyzed microbiologically following the methods of Ciafardini et al. [12]. The yeasts and molds were evaluated using Petri dishes with MYGP agar medium containing: 3 g yeast extract (Biolife, Milan, Italy), 3 g malt extract (BBL, Cockeysville, MD, USA), 2.5 g soy peptone (Biolife), 2.5 g bacto tryptone (BBL), 10 g D-glucose (Merck, Darmstadt, Germany), 1000 mL distilled water, and pH 7 as described by Kurtzman and Fell [14]. This medium was supplemented with sodium propionate (2 g/L) and tetracycline (20 mg/L) in order to inhibit growth of molds and bacteria respectively. 200 *μ*L of the above decimal dilution was plated in triplicate, onto Petri dishes with the medium using the spread plating techniques. The total yeasts colony form units (CFU) were counted after 5 days of incubation at 30°C whereas the total molds CFU were evaluated after 7 days of incubation at 28°C. The total coliform bacteria CFU were evaluated on Violet Red Bile Agar (VRBA, cod. CM0107 Thermo Fisher Diagnostics, MI, Italy). The medium was inoculated with 200 *μ*L of the decimal dilution, then the Petri dishes were incubated 16 h under aerobic conditions at 37°C and the CFU were recorded from all samples [15].

### 2.3. E. coli Isolation

A series of single colonies from all the analyzed samples that appear purple on the VRBA medium were isolated for* E. coli* identification according to the European Commission Regulation UNI EN ISO 9308 [16] and tested separately for Gram stain, cytochrome oxidase activity, and indole production. Moreover, the selected single colonies were transplanted into the Tryptic Soy Agar medium (Sigma-Aldrich cod. 22091) containing 15 g casein peptone, 5 g soya peptone, 5 g NaCl, 20 g agar, 1000 mL distilled water, and pH 7. After 24 h of incubation at 36°C, the bacterial cultures were used for the cytochrome oxidase test. The enzymatic reaction was assessed after transferring the bacterial cultures onto the Petri dish on some pieces of paper filters that had been moistened with an aqueous solution containing 1% (w/w) of N, N, N′, N′-tetramethyl-*p*-phenylenediamine dihydrochloride (Sigma-Aldrich cod. 87890). The appearance or not of the blue color on the filter paper was recorded after 5 min of incubation at room temperature. The indole production was evaluated after having transferred part of the bacterial cultures in test tubes equipped with a screw cap containing 10 mL of Tryptophan Culture Broth (Sigma-Aldrich cod. 09136) with the following composition: 10 g casein enzymatic hydrolysate, 5 g NaCl, 1 g DL-tryptophan, 1000 mL distilled water, and pH 7.5. After 24 h of incubation at 43°C, 0.5 mL of the Kovac's reactive (4-dimethylamino-benzaldehyde solution, Sigma-Aldrich cod. 3381) was added to each test tube. The positive reaction occurred after 5 min of incubation at 43°C with the appearance of a red color on the top of the substrate. The commensal* E. coli* ATCC 25922 was used as a positive denominator, while as a negative* Enterococcus faecalis* ATCC 19433 was used. The selected cultures of commensal* E. coli *cytochrome oxidase-negative and indole-positive were confirmed with the API 20E (bioMérieux, France) test and the PCR as reported by Omar et al. [17]. Then three* E. coli *cultures isolated, respectively, from the wash water of each olive variety were used in the laboratory inoculation trials described below.

### 2.4. Laboratory Inoculation Trials

The inoculation trials were carried out in the laboratory in order to evaluate the survival of some olive-born coliform bacteria in olive oil characterized by a different total polar phenols' content. The coliform bacteria used in this study were represented by three commensal* E. coli* strains that had been isolated as, aforementioned, using the wash water samples from each olive variety. The* E. coli* cultures were grown in 1 L flasks containing VRB Broth, after 24 h of incubation at 37°C under aerobic conditions, the bacteria cells were separated by centrifugation at 5,000 g for 10 min using Hettich centrifuge, mod. Universal 32 (Hettich Instrument, Tüttlengen, Germany) and then used for the inoculation trials. Virgin olive oil used in the trials came from the extraction process of the Taggiasca olives collected at a different degree of ripeness which affected the polyphenols content of the oily fraction of the fruits. The trials were then carried out using three types of virgin olive oils characterized by a total polyphenols content equal, respectively, to 28, 110, and 372 mg of caffeic acid equivalent per kg of product. The samples of olive oil with the different total polyphenols content were sterilized through microfiltration with a nitrocellulose filter with a porosity of 0.45 *μ*m (Minisart NML-Sartorius, Göttingen, Germany), and a mass equal to 2,000 mL was transferred into sterile empty Pyrex flasks and inoculated with 2 g of* E. coli* biomass with 40% humidity, suspended in 20 mL of sterile olive oil reaching a final concentration equal to 0.1% (w/v). The inoculated* E. coli* biomass was prepared by collecting, in equal proportions, the biomass of the aforementioned* E. coli* strains isolated, respectively, from the wash water of the Lavagnina, Taggiasca, and Leccino olive variety. After 1 min of agitation with a vortex, all the inoculated olive oil samples were stored in a dark place at room temperature for 30 days. The trials were accomplished using uninoculated olive oil samples (control) and three repetitions. The survival of the* E. coli* inoculated in the virgin olive oil samples with different total polyphenols content was assessed by the microbiological analysis of 10 mL of oil samples, taken after every 5 days, during the storage time, using the same procedure as described above. At the end of the storage time, the* E. coli* cells were recovered from the residual inoculated virgin olive oil samples and used for the ultrastructural cells observation and the bacterial protein assay as described below.

### 2.5. Polyphenols' Interaction with the Bacterial Protein

The polyphenols' interaction with the bacterial proteins was studied by analyzing the protein extracted from* E. coli* which had been stored for 1 month in virgin olive oil samples containing, respectively, 28 and 372 mg of caffeic acid equivalent per kg. The bacterial cells were separated by the oily fraction through the centrifugation at 7,000 g for 15 min and then suspended in 10 mL of 0.1M phosphate buffer, pH 7.2, agitated vigorously for 1 min and finally centrifuged as before, to allow for the complete elimination of the oily residues. The bacterial biomasses prepared separately from each type of inoculated olive oil sample as well as the bacterium used originally as inoculum were used for the electrophoretic analysis. The crude bacterial extract was prepared from each inoculated olive oil sample, using 2.5 mL of 0.5 M Tris HCl buffer, pH 6.7, with 0.8 g of bacterial biomass. The proteins were extracted by breaking the cell walls with a Sonifier apparatus (Branson B 20 Sonifier). The power was 60 W and the cells were submitted to a cycle of 5 s of sonication for a total period of 15 min. At the end of each period of sonification a part of the bacterial crude extract was withdrawn, centrifuged at 10,000 g for 5 min and tested with the Biorad protein assay dye (Biorad Laboratories, Munich, Germany) with the aim of evaluating the amount of total proteins present in the liquid fraction. On average, the protein concentrations of the stocks of crude bacterial extracts, used for the following experiments, varied between 60.20 and 61.30 *μ*g of bovine albumin equivalent per mL. The analysis was performed using sodium dodecyl sulphate polyacrylamide gel electrophoresis (SDS-PAGE) according to Laemmli [18]. The analysis was accomplished with a PROTEAN II maxi cell (Biorad, Richmond, CA, USA) suitable for 160x200 mm gels. The gels contained 12% polyacrylamide and the electrophoresis was run with constant current as suggested by the manufacturer. The SDS-PAGE gels were stained with Coomassie Brilliant Blue R-250 (Sigma-Aldrich, Milan, Italy). The low-weight calibration Kit from Pharmacia LKB-Biotechnology (Piscataway, NJ, USA) was used as a standard molecular mass.

### 2.6. SEM Observation

The SEM observations were carried out using the bacterial cells recovered from the inoculated olive oil samples characterized by 28, 110, and 372 mg per kg of total polyphenols as reported in the trials described above. Volumes of the inoculated olive oils equal to 10 mL were taken after 30 days of incubation and were filtered using a nitrocellulose membrane filter with a porosity of 0.45 *μ*m. The filters with the* E. coli* bacteria were then cut into several pieces, suspended in 10 mL of sterile physiological solution with 0.9% NaCl, and mixed slowly with a mixer for 5 min. The bacteria suspended in the liquid fraction were then collected by centrifugation at 7,000 g for 5 min. The biomasses were fixed in 1 mL of 3% glutaraldehyde (v/v) in 0.1M phosphate buffer, pH 7.2 for 12 h. The samples were rinsed in the same buffer three times and then dehydrated (twice for each solution) in a graded ethanol series (20, 40, 60, 80, 95, and 100%) for 10 min each with a final wash in acetone for a better CO_2_ substitution during the dehydration procedure, at a pressure of 1200 bars. Subsequently, all samples were dried in a CO_2_ critical point dry (Emitech K850) and then covered with palladium gold in Emitech K550 before SEM observation.

### 2.7. Statistical Analysis

A priori one-way analysis of variance, using Tukey's honest significant differences test, was performed using a statgraphics computer program (Statgraphics, version 6, Manugistics, Inc., Rockville, MA), and any values which were different at p < 0.01 were recorded.

## 3. Results and Discussion

**Figure 1:**
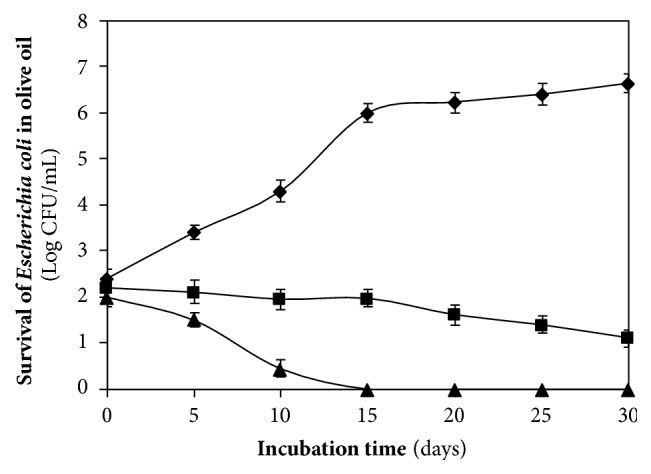
Survival of* E. coli* inoculated in extra virgin olive oil containing 28 (♦), 110 (■), and 372 (▲) mg of total polar phenols per kg of product.

**Figure 2:**
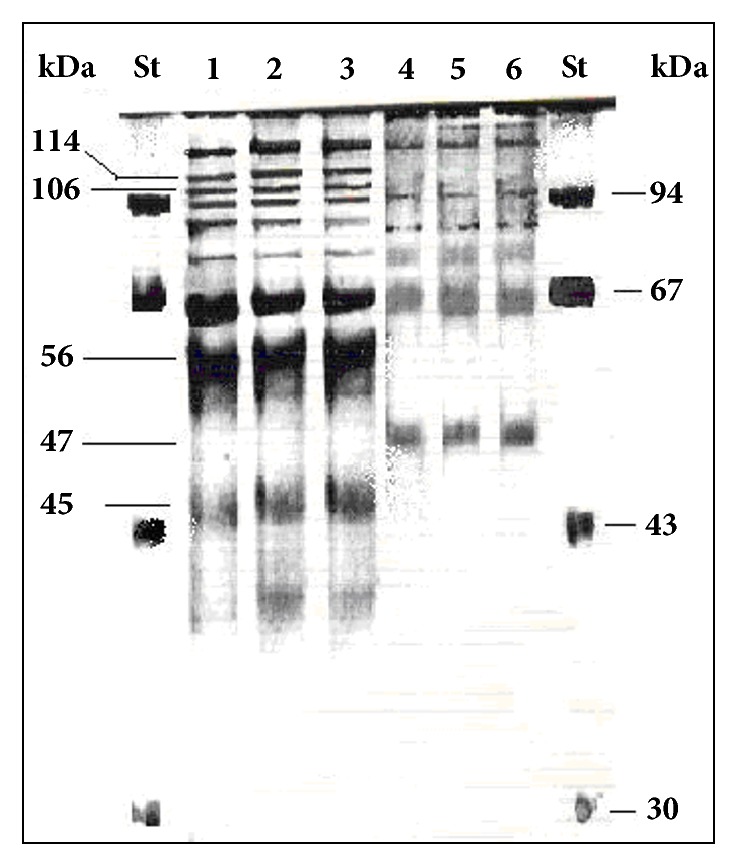
SDS-PAGE of protein crude extract from cells of* E. coli *stored one month in olive oil with different phenolic compounds concentration. 1, SDS-PAGE of total proteins from untreated* E. coli* (control). 2, 3, SDS-PAGE of total proteins from* E. coli* stored in olive oil samples with low total polar phenols concentration equal to 28 mg caffeic acid equivalent per kg. 4, 5, 6, SDS- PAGE of total proteins from* E. coli* stored in olive oil samples with high polar phenols content equal to 372 mg caffeic acid equivalent per kg. St, molecular standard weigh (kDa). The arrows indicate the differences between the SDS-PAGE of protein from* E. coli* stored in olive oil compared to the control.

**Figure 3:**
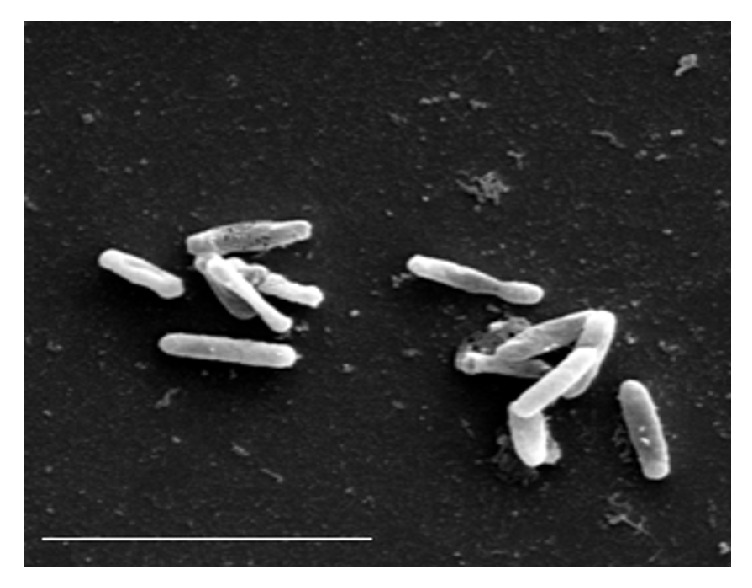
SEM observation of undamaged one-month* E. coli* cells stored in olive oil with 28 mg of total phenolic compounds per kg (bar=4 *μ*m).

**Figure 4:**
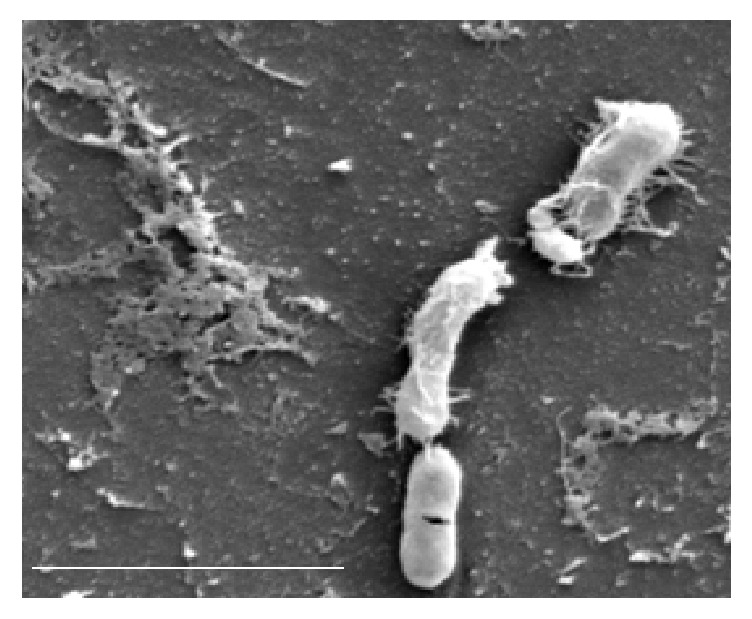
SEM observation of damaged one-month* E. coli* cells stored in olive oil with 110 mg of total phenolic compounds per kg (bar=4 *μ*m).

**Figure 5:**
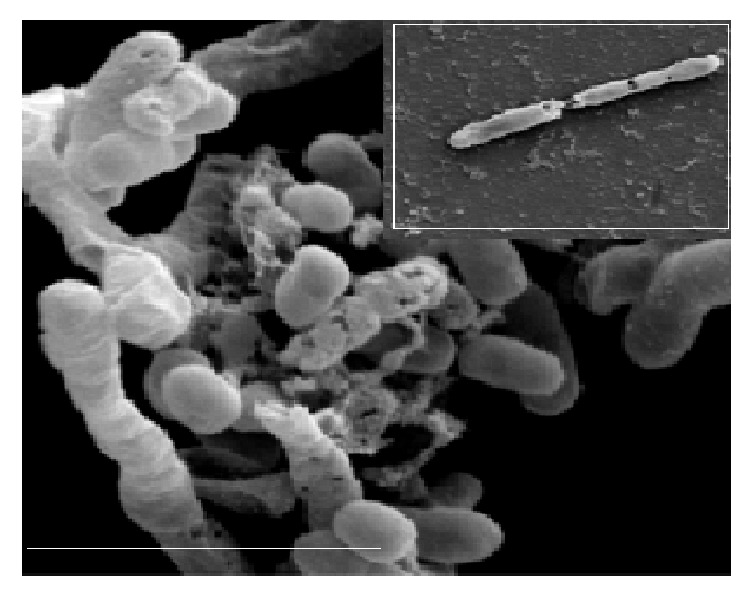
SEM observation of damaged one-month* E. coli* cells stored in olive oil with 372 mg of total phenolic compounds per kg (bar=4 *μ*m).

The microbiological analysis of the wash water, kneaded paste, and the extra virgin olive oil produced by three olive varieties showed the presence of yeasts and molds in all the analyzed samples, whereas coliform bacteria were found in the wash water produced, respectively, by the Lavagnina, Taggiasca, and Leccino variety, but were absent in the kneaded paste and the olive oil extracted by the same olive varieties. On the other hand, in all the wash water samples, the number of coliform bacteria CFU per mL was much higher compared to those of the yeasts and molds, while no significant differences were observed between the CFU number of the yeasts and molds detected in the samples of three olive varieties analyzed (Table 1). The total polar phenols content of the extracted extra virgin olive oil varied between 205 and 250 mg caffeic acid equivalent per kg, according to the olive variety, while the total polar phenols content of the kneaded paste, in turn, varied from 1,200 to 1,800 mg caffeic acid equivalent per kg (Table 1). The study of the distribution of coliform bacteria from the olives' carposphere in different substrates produced in the mills during the oil extraction process indicated, for the first time, that the kneaded paste exerts a strong selective pressure on the survival of the coliform bacteria originating from the fruits. Moreover, the results reported in Table 1 show yeasts, molds, and a large number of coliform bacteria from the carposphere of the fruits to be present in wash water where the polar phenols were absent, while in the subsequent products containing polar phenols like the kneaded paste and the extracted olive oil, it is still possible to note the presence of a significant number of yeasts and molds but not of coliform bacteria. This behaviour observed for all the varieties of olives studied can be attributed to the different phenolic compounds content of the oil mill products that, among other cause, interfere with the viability of the different microorganisms. Novelty of these results is important not only from a technological point of view, since, as known, the malaxation affects the chemical composition and sensorial characteristics of the product [19, 20], but mainly in terms of hygiene. In fact, the malaxation of the paste in the mills rich of phenolic compounds (Table 1) represents a process where the natural sanitization of the product occurs through the reduction or complete destruction of the viable coliform bacteria from the fruits and other sources. On the contrary, a very different behaviour has been recorded in the newly produced olive oil. In fact, the survival of the bacterium* E. coli *artificially inoculated in virgin sterile olive oil, characterized by an increasing content of phenolic compounds, varied according to the phenolic concentration (Table 2). The olive oil polar phenols inhibited the survival of the bacterium when their concentration was greater than 110 mg of caffeic acid equivalent per kg of product, whereas below this value no inhibitory effect was detected (Figure 1). In detail, when the bacterium* E. coli* was inoculated in the olive oil characterized by a high polar phenols content, equal to 372 mg caffeic acid equivalent per kg of product, the CFU per mL of oil decreased drastically already during the first few days of incubation, while they were completely absent after 15 days of storage. In turn, in the olive oil samples with 110 mg of caffeic acid equivalent per kg, the number of the* E. coli *living cells remained constant in the first 15 days of incubation and then gradually shrank to 1.5 Log at the end of storage. However, from Figure 1 it is possible to note that* E. coli* can grow in the polyphenol-poorly olive oil. In fact, when the bacterium was inoculated in the olive oil with low polar phenols content, equal to 28 mg caffeic acid equivalent per kg, the number of the* E. coli* CFU per mL increased three times during the first 15 days of incubation, reaching more than 6 Log CFU per mL after 1 month of incubation. These results indicate that not all oils classified as virgin olive oil or refined olive oil had similar bactericidal effects and are considered unsuitable for the survival of coliform bacteria, since their bioactivity depends on the concentration of the total polar phenols as well as the profile of the phenolic compounds. The edible phenolic-poor olive oils are represented by refined olive oils, where these compounds are lost during the chemical treatments of the product as well as some virgin olive oils [21]. Several studies have shown that the concentration of the total polar phenols of virgin olive oil varies with cultivars, ripeness, climate conditions, irrigation, and extraction process [10, 22–24]. The experiments carried out with the* E. coli* stored in virgin olive oil containing different total polyphenols content demonstrated that the phenolic compounds when they are present in sufficient concentrations are able to react with the proteins of the bacterium. In fact, the total proteins extracted from* E. coli* stored for 1 month in olive oil with 372 mg caffeic acid equivalent per kg distinguished themselves from those extracted from the same bacterium that was not suspended in olive oil (control) as well as those stored in virgin olive oil with low level of polar phenols equal to 28 mg caffeic acid equivalent per kg, by type of electrophoretic pattern including between 45 and 114 kDa weight obtained with the SDS-PAGE analysis (Figure 2). Research carried out until now on other species of bacteria has shown that the phenolic compounds of olives bond tightly to the cell wall thus damaging them [25]; nevertheless it is as yet unknown with which part of the bacterial cells the polyphenols of olive oil react. In any case, the results recorded in Figure 2 help to clarify the mechanism of the antimicrobial action carried out by the polyphenols as they have a strong protein-denaturing activity as shown by the SDS-PAGE analysis. SEM observations confirmed the results of the electrophoretic analysis since no serious ultrastructural damage on the cell walls of* E. coli* stored in olive oil with 28 mg per kg of total polar phenols were recorded (Figure 3). In turn, the bacterial cells suspended in the virgin olive oil with a total polar phenols concentration equal to 110 mg per kg of product showed single breakages which were often located at the centre of the cells (Figure 4), while the SEM ultrastructure of* E. coli* inoculated in the polar phenols-rich olive oil, equal to 372 mg caffeic acid equivalent per kg, showed a generalized series of slots, distributed over the entire cell wall of many bacteria (Figure 5). On the basis of these results, it seems that the damage observed on the cell wall ultrastructure was more evident when the bacterial cells were inoculated in olive oil samples characterized by a total polar phenols content greater than 110 mg per kg (Figures 4 and 5). On the contrary, the bacterial cells suspended in olive oil with a low total phenol compounds content were not damaged (Figure 3). Comparing the results of SDS-PAGE analysis of the* E. coli* proteins reported in Figure 2 with the SEM ultrastructural modifications (Figures 4 and 5), it is possible to hypothesize that the above ultrastructural modifications can be attributed to olive oil polar phenols that bind to the cell wall components. Another research carried out so far has demonstrated that the phenolic compounds react permanently with the walls of the yeast* Candida parapsilosis* according to their concentration in the inoculated olive oil [6]. In the bacteria, an SDS-PAGE analysis performed with crude extract of* Xanthomonas campestris* have confirmed that olive oil polyphenols are able to react with the bacterial protein, producing a very strong protein-cross-linking and protein-denaturing effect [5]. However, the presence of some hydrolysed cells, shown in Figure 5, indicate that the olive oil phenolic compounds inhibited the survival of the bacterium also through other mechanisms. In fact, the alterations of cell walls of* E. coli* observed with the SEM are compatible with the oxidative stress caused by the accumulation of hydrogen peroxide and Reactive Oxygen Species (ROS) produced by certain concentrations of olive oil phenolic compounds, which can act as antioxidants or prooxidants depending on the environmental conditions, interaction with metal, structural changes, concentration, and exposure to microorganisms [26, 27]. Zanichelli et al. reported that the inhibition of* Staphylococcus aureus* by an olive oil phenolic compound known as oleuropein is largely due to hydrogen peroxide [28], while Taleb et al. demonstrated that the total polar phenols extracted from the Date syrup suppress the growth of* E. coli* at a Minimum Inhibitory Concentration (MIC) of 30 mg per mL and that hydrogen peroxide was produced at lethal but not sublethal concentrations of total polar phenols [29]. All these results highlight that the antibacterial activity of polar phenols is mediated through hydrogen peroxide generation in inducing oxidative stress in bacteria as well as interaction with bacterial proteins.

## 4. Conclusion

Coliform bacteria consist of both nonpathogen commensal and pathogen species widely in different habitats including the carposphere of the olives processed in the mills. The contaminating coliform bacteria are destroyed in the oil mill during the malaxation process of the paste that is usually richer in phenolic compounds compared to the extracted olive oil. This technological process is important because it prevents the contamination of the newly produced olive oil by the coliform bacteria originating from the fruits, the operators, or the mills. However, the newly extracted olive oil can be contaminated later by the coliform bacteria, which can survive and reproduce in this habitat if the total phenolic compounds content is too low. The results demonstrate that olive oil polar phenols can prevent the survival of the* E. coli *present in the product after the extraction process, only in the presence of sufficient concentrations, which in our study was higher than 110 mg of caffeic acid equivalent per kg. This finding needs further studies since in an era of antibiotic resistance, the development of new strategies to fight unwanted food bacteria is promising way for the future. In fact, the novelty of these interesting findings lies in the fact that, for extra virgin olive oil, which, according to current regulations, can be extracted only by mechanical processes without any chemical treatments, it is possible to improve the hygienic properties of the product by intervening on the malaxation phase and on the phenolic content of the product. On the basis of the results obtained by the trials, it is possible to assert that the use of good quality edible virgin olive oil, characterized by a sufficient total phenolic compounds content, does not generally constitute a risk factor for the health of consumers.

## Figures and Tables

**Table 1:** Chemical and microbiological analysis of different substrates from oil mills during the olive oil extraction process of three varieties.

Olive variety	Wash water				Kneaded paste				Olive oil			
	Total phenols^*∗*^	Coliforms^*∗∗*^ bacteria	Yeasts	Molds	Total phenols	Coliforms bacteria	Yeasts	Molds	Total phenols	Coliforms bacteria	Yeasts	Molds
Lavagnina	-	4.3 ± 0.4^a^	2.5 ± 0.3^b^	1.8 ± 0.1^bc^	1,200 ± 80	-	2.0 ± 0.2^b^	2.2 ± 0.1^b^	205 ± 10	-	1.5 ± 0.1^c^	1.2 ± 0.2^c^
Taggiasca	-	4.5 ± 0.7^a^	2.7 ± 0.2^bc^	2.8 ±0.2^bc^	1,500 ± 120	-	3.3 ± 0.3^b^	2.2 ± 0.1^c^	230 ± 12	-	2.8 ± 0.3^bc^	2.0 ± 0.1^c^
Leccino	-	6.0 ± 0.6^a^	2.5 ± 0.2^b^	0.8 ± 0.1^c^	1,800 ± 155	-	2.2 ± 0.1^b^	0.9 ± 0.08^c^	250 ± 14	-	1.6 ± 0.2^bc^	1.5 ±0.2^bc^

^*∗*^mg caffeic acid equivalent/Kg (mean ± standard deviations, n = 6). ^*∗∗*^ Log UFC/g (mean ± standard deviation, n = 6): values in row having different letters are statistically different from one another at p < 0.01.

**Table 2:** Analytical indices of three types of olive oil from Taggiasca variety used in the laboratory inoculation trials.

Inoculated virgin olive oil type	Total phenols (mg caffeic acid equivalent/kg)	Free fatty acid (% oleic acid)	Peroxide value (meqO_2_/kg)	K_232_	K_270_	ΔK
A	372 ± 12	0.30 ± 0.01	8.75 ± 0.07	1.705 ± 0.02	0.100 ± 0.009	-0.005 ± 0.001
B	110 ± 4.50	0.37 ± 0.02	10 ± 0.11	1.791 ± 0.03	0.110 ± 0.008	-0.004 ± 0.000
C	28 ± 2.80	0.45 ± 0.03	18 ± 0.73	1.845 ± 0.02	0.117 ± 0.005	-0.002 ± 0.000
Limit for the “extra virgin” merceological class		≤ 0.80	≤ 20	≤ 2.50	≤ 0.22	≤ 0.010

*∗*Mean ± standard deviation, *n* = 3.

## Data Availability

The original data used to support the findings of this study are available from the corresponding author upon request.
